# Assessing the climate change mitigation potential from food waste composting

**DOI:** 10.1038/s41598-023-34174-z

**Published:** 2023-05-10

**Authors:** Tibisay Pérez, Sintana E. Vergara, Whendee L. Silver

**Affiliations:** 1 Department of Environmental Science, Policy, and Management, University of California, Berkeley, CA 94720 USA; 2 Centro de Ciencias Atmosféricas y Biogeoquímica, Instituto Venezolano de Investigaciones Científicas, Caracas, Aptdo 1020A, Venezuela; 3 Department of Environmental Resources Engineering, Humboldt State University, 1 Harpst Street, Arcata, CA 95521 USA

**Keywords:** Biogeochemistry, Environmental sciences, Climate-change mitigation, Carbon cycle, Biogeochemistry, Climate-change policy, Sustainability

## Abstract

Food waste is a dominant organic constituent of landfills, and a large global source of greenhouse gases. Composting food waste presents a potential opportunity for emissions reduction, but data on whole pile, commercial-scale emissions and the associated biogeochemical drivers are lacking. We used a non-invasive micrometeorological mass balance approach optimized for three-dimensional commercial-scale windrow compost piles to measure methane (CH_4_), nitrous oxide (N_2_O), and carbon dioxide (CO_2_) emissions continuously during food waste composting. Greenhouse gas flux measurements were complemented with continuous oxygen (O_2_) and temperature sensors and intensive sampling for biogeochemical processes. Emission factors (EF) ranged from 6.6 to 8.8 kg CH_4_–C/Mg wet food waste and were driven primarily by low redox and watering events. Composting resulted in low N_2_O emissions (0.01 kg N_2_O–N/Mg wet food waste). The overall EF value (CH_4_ + N_2_O) for food waste composting was 926 kgCO_2_e/Mg of dry food waste. Composting emissions were 38–84% lower than equivalent landfilling fluxes with a potential net minimum savings of 1.4 MMT CO_2_e for California by year 2025. Our results suggest that food waste composting can help mitigate emissions. Increased turning during the thermophilic phase and less watering overall could potentially further lower emissions.

## Introduction

Over one-third of global food production is estimated to enter the waste stream, where it contributes to greenhouse gas (GHG) emissions^1,2^. Average lifecycle GHG emissions from food loss and waste (FLW) are estimated to be 124 g CO_2_e per capita globally and 315 g CO_2_e per capita in high income nations^3^. In the U.S., FLW ranges from 73 to 152 MMT/y or 223 to 468 kg per capita annually^4^ (MMT = million metric tons). The most important FLW management pathways in the country are landfilling (56%), controlled combustion (12%), co-digestion/anaerobic digestion (8%), and sewer/water treatment (6%), with composting representing only about 4.1%^5^. Food waste has the largest fraction of decomposable degradable organic carbon (C) when comparing to other organic waste (wood, paper and yard trimming), driving the highest rate constant for GHG production in landfills (2708 kg CO_2_e/dry t)^6^. Landfills are the third largest source of CH_4_ emissions in the U.S. GHG inventory, due primarily to the anaerobic decomposition of C-rich organic waste^7–10^. Life cycle assessment (LCA) studies suggest that considerable GHG savings could be achieved if organic waste was managed via aerobic composting or anaerobic digestion, rather than conventional management strategies^11^.

Composting is a form of managed organic matter decomposition. Typical commercial-scale aerobic composting practices include in vessel, windrow and forced aerated static piles^12^. In the U.S., composting is generally conducted in windrows and static piles in open-air facilities^13^. Organic matter decomposition in windrows and static piles passes through four discrete, thermally defined phases during composting. Early-phase decomposition is characterized as mesophilic (25–40 °C) with the conversion of the most easily degradable material into carbon dioxide (CO_2_) and microbial products. Increasing microbial activity and associated temperatures leads to a thermophilic phase (40 to 65 °C). High rates of microbial activity during this phase can result in oxygen (O_2_) depletion and the prevalence of anaerobic microbial processes such as methanogenesis^14,15^. Piles are mechanically turned regularly during the composting process to limit the development of anaerobiosis. As the amount of easily degradable material declines and the formation of more recalcitrant organic material increases, decomposition slows, and temperatures begin to cool during the second mesophilic phase. The final phase of composting is termed maturation and is characterized by the decline in bacterial biomass and increase of fungi as temperatures return to ambient levels^14^.

Physicochemical parameters are important drivers of GHG fluxes during organic matter decomposition and are likely to affect emissions during composting. For example, the ratio of C to nitrogen (N) determines microbial N availability during decomposition; higher C:N ratios are generally found at the beginning in comparison to the end of the composting process^16^. Substrate pH, moisture, O_2_ concentrations, and porosity can affect patterns and rates of soil organic matter and litter decomposition^17^, and are also likely to drive decomposition of organic waste^16,18^. Starting organic matter constituents in compost piles, called feedstocks, differ in their C and nutrient content, moisture levels, cellulose content, and proportion of complex organic molecules such as lignin^19^. Food waste is particularly C- and nutrient-rich and moist, facilitating the production and emissions of GHG fluxes in landfills. Food waste has the highest rate constant for CH_4_ generation in landfills due to its large fraction of labile C^9^. High C and N concentrations of food waste could potentially drive high nitrous oxide (N_2_O) emissions during the composting process, particularly if the pile is moist and well aerated. Aerated conditions can stimulate nitrification in compost piles^20^. Short-term anaerobic events or microsites with high moisture content can result in nitrifier-denitrification and canonical denitrification producing N_2_O. However, data on patterns and the associated drivers of trace gas emissions from commercial-scale food waste composting are lacking. Understanding how spatial and temporal patterns in biogeochemical dynamics relate to GHG emissions during the composting process is essential for predicting GHG fluxes from composting.

In California, the largest food producing state of the U.S., organic waste represents approximately 34% of total solid waste disposal, with discarded food making up 44% of the organic matter contribution^21^. Most of the 5.3 MMT of food waste generated in California is landfilled, with the remainder processed through alternative management such as composting, recycling, incinerating and anaerobic digestion. Silver et al.^22^ suggested that diverting the largest organic waste streams in the state of California from landfilling to composting could potentially result in net GHG reductions compared with current management practices. Additional GHG savings could be achieved by using compost as an alternative to inorganic or high-emitting organic fertilizers (e.g. livestock manure), and from increased soil C sequestration following compost amendments to soils^23–26^. California recently launched an aggressive policy of 75% diversion of organic waste from landfilling to alternative management by 2025 (SB 1383)^27,28^. Understanding the patterns and drivers of GHG emissions from composting is essential to determine the potential for climate change mitigation from these types of policy changes^27^.

Determining emissions from commercial-scale composting is challenging. Most measurement approaches such as static chambers, enclosed piles, and short-term assays introduce errors associated with changes in GHG drivers and can miss hot spots and hot moments of emissions^22^ (see Supplementary Table S2 online). To accurately estimate real-world conditions, compost emissions should be measured continuously during the composting process, capture the entire period from pile formation to finished compost, and follow emissions during typical commercial-scale procedures. Very few studies have met these criteria, and the few that have used approaches that are not directly comparable^18,29–33^. Micrometeorological approaches have been proposed as a means to better quantify GHG emissions during composting. Micrometeorological approaches can be implemented in the field and are noninvasive, enabling the study of the entire composting process while avoiding the problems typically associated with enclosure of compost piles such as changes in gas diffusion, moisture or temperature^34^. Micrometeorological methods also have the potential to make continuous measurements, reducing the chances of missing hot spots or hot moments of emissions and thus producing a more realistic estimate of greenhouse gas emissions^33,35–39^. In this study, we used a micrometeorological mass balance (MMB) approach to measure continuous CO_2_, N_2_O, and CH_4_ emissions during commercial-scale food waste composting in California, USA. Our optimization of the micrometeorological mass balance approach facilitates GHG measurements across a three-dimensional structure with high resolution in space and time and can be used at a large scale. The combination of land–atmosphere fluxes and continuous sensing within the pile allowed us to intensively sample the compost environment to determine both the patterns and drivers of GHG emissions during the entire food waste composting process^13^. We hypothesize that the GHG emission factors (EF) associated with the composting process are smaller than those derived from conventional landfill disposal of food waste. Based on the measured physicochemical drivers of GHG fluxes, we determine the potential GHG savings for California by the year 2025 and biogeochemical factors that would further mitigate GHG from this management practice.

## Methods

The experiment was conducted at the West Marin Composting Facility in Nicasio, California (38°05′14.9"N 122°42′26.0"W). We established one windrow pile of approximately 15 × 4 × 2 m (length, width, height) (Aug. 17, 2018). The experiment ran for 80 d until the material was fully composted based on state guidelines^32^. The pile composition was 34.3% w/w (22% v/v) food waste with yard debris as bulking agent (Table 1).

**Table 1:** Specifications of the food waste compost pile.

Material	Composition	Weight (Mg)	Density (kg/m^3^)	Volume (m^3^)
Food waste	Vegetables and fruits (~ 80%); flour and bread (~ 10%); meat (~ 10%)	14.7	783	24.8
Yard debris	Garden trimmings, wood chips	28.1	415	67.7
Percentage food waste		34.3% w/w^a^		22% v/v

^a^The selected ratio 1:2 (food waste: bulking agents) was determined by best practices adopted by the compost facility and was based on the specific feedstocks C/N values that produced an initial compost pile C/N of 30. This obtained pile C/N was within the recommended range (25 to 40) (see Rynk et al.^40^ for calculation details).

Food waste was derived from Marin County farmer’s markets and restaurant organic waste. Contaminants (glass, metal, plastic, etc.) were removed manually, the material was mixed with the bulking agent, and mechanically turned with a windrow turner^41^.Water was added at the beginning of the composting process (9464 L), and on d 18 (946 L), 24 (1893 L), 31 (846 L), 52 (2650 L), and 66 (5243 L) based on the standard commercial composting practices^42^.

**Figure 1:**
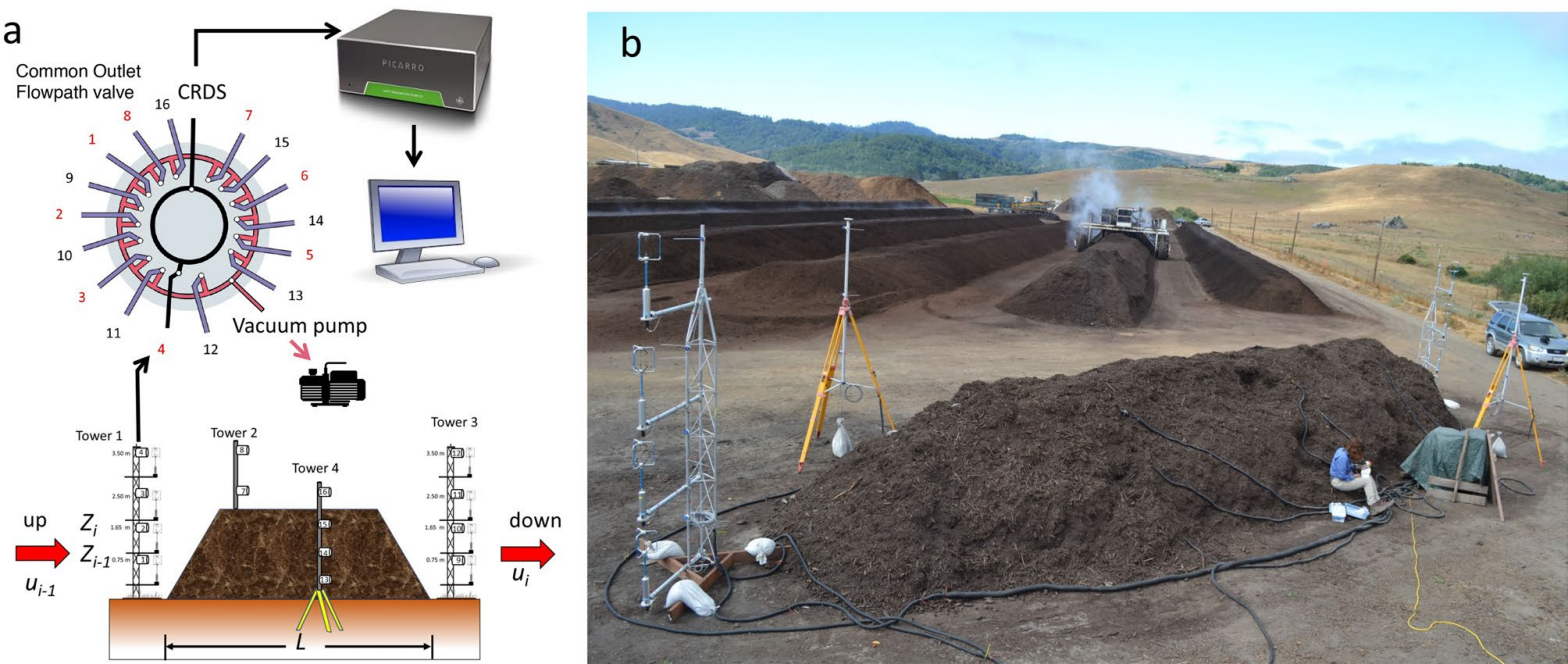
(**a**) Diagram of micrometeorological mass balance method experimental set up. (**b**) Field layout of the experimental compost pile (picture credit: Kris Daum).

Greenhouse gases fluxes were measured using an adaptation of the MMB method applied by Wagner-Riddle et al.^38^ Four towers were placed around the pile. Each tower was outfitted with four Teflon gas sampling tubes (1/8″ O.D.) at heights of 0.75 m, 1.65 m, 2.50 m, and 3.50 m, for a total of 16 gas sampling inlets. Each sampling inlet had a 0.45 mm membrane filter to prevent particle interference and moisture saturation. Atmospheric CH_4_, CO_2_, and N_2_O concentrations were measured continuously using a cavity ring-down spectrometer (CRDS) (G2308, Picarro, Santa Clara, CA). A low-pressure common outlet flowpath selector (EUTA-VLSF8MWE2, Vici, Houston, TX) with 16 tube sampling ports was connected to the sample inlet tubes placed at each height on the towers (Fig. 1a). Air constantly flowed from the sampling inlets through the common outlet selector connected to an external vacuum pump (Fig. 1a). By maintaining continuous airflow through the sample lines, we guaranteed that when a sample stream was ready for analysis, the air mass would be representative of the tower’s selected sampling inlet height. When a sample stream was selected for GHG concentration analysis, the air was routed to the CRDS where GHG concentrations were measured at 1-min intervals (Fig. 1a).

Two of the towers were located length wise (Fig. 1a) and instrumented with four 3D sonic anemometers (Gill Wind Master Pro, Gill Instrument, Lymington, England) each, installed at the same heights as the gas sample inlet ports to measure meteorological variables (wind speed, wind direction, and sonic temperature) continuously (every 15 s, 1 Hz) during the entire composting process. Air samples at each of the four sampling heights were drawn in successive pairs from opposite towers to minimize the time elapsed between upwind and downwind samples and maximize the likelihood that micrometeorological conditions remained similar during both sampling periods. With this technique, we measured fluxes continuously alternating lengthwise from tower 1 (T1) to tower 3 (T3) and widthwise from tower 2 (T2) to tower 4 (T4). For example, gas samples collected from inlet 1 (from height 0.75 m at T1) were followed by gas collection at the same height in the opposite tower (sample inlet 9, T3) (Fig. 1a). This process was continued until the highest sampling port (3.5 m) was reached, and then the cycle restarted at the lowest height again.

The flux equation assumes that the turbulent diffusive flux is negligible and can be approximated by^38^ Eq. (1):

1 $$flux = \frac{1}{L}\mathop \smallint \limits_{0}^{\infty } \overline{u}_{z} (\overline{c}_{z,out} - \overline{c}_{z,in} ) dz$$

where L (m) is the linear distance between the upwind and downwind measuring towers (fetch) and ${\overline{u} }_{z}$, ${\overline{c} }_{z,d}$ and ${\overline{c} }_{z,u}$ are the mean horizontal wind speed (m/s) at each sample height *z*, and gas concentrations for downwind and upwind towers (mg GHG/m^3^), respectively. We used two integration methods: a trapezoidal rule and a fitted spline function. The concentration difference (*ΔC*_*i*_) at a height *z*_*i*_ is given by the difference between the GHG concentration at the downwind (*C*_*i*-*down*_) minus the upwind (*C*_*i*-*up*_) sides of the pile (Eq. 2). The flux between heights *z*_*i*_ and *z*_*i-1*_ is calculated by the average of the concentration difference multiplied by the respective mean horizontal wind speeds (*ū*_*i*_*. ΔC*_*i*_* and ū*_*i-1*_*. ΔC*_*i-1*_). The mean is then integrated over the two sampling heights (*z*_*i*_ and *z*_*i-1*_) (Eq. 3) and divided by the fetch (*L*).

2 $$\Delta C_{i} = C_{i - down} - C_{i - up}$$

3 $$flux = \frac{1}{L}\mathop \sum \limits_{i = 1}^{n} \left( {(z_{i} - z_{i - 1} )\begin{array}{*{20}c} {\underline{{\left( {\overline{u}_{i} \overline{\Delta C}_{i} + \overline{u}_{i - 1} \overline{\Delta C}_{i - 1} } \right)}} } \\ 2 \\ \end{array} } \right)$$

This method neglects the horizontal turbulent diffusive term, and thus it could overestimate fluxes^39^. An empirical test of potential overestimation for CH_4_ emissions showed that it was approximately 5% when using both fast response anemometers and concentration methods^43^. The instrumentation used here met the requirements to minimize any potential overestimation^43,44^, and thus we did not apply a correction factor.

This method assumes that the vertical flux is negligible. This is achieved when the highest sampling port is placed above the air mass layer where the dominant horizontal flux takes place. By convention, this height is determined by dividing the source’s longest horizontal distance by ten^38^. Here, the diagonal length of the rectangular base of the pile was 15.5 m and the selected height of the top sampling port was 3.5 m, which in theory is large enough to guarantee that no significant vertical flux occurred. For details about the method optimization refer to supplementary material.

We continuously monitored temperature (CS616, Campbell Scientific, Logan, Utah, USA) and O_2_ concentrations (SO-110, Apogee Instruments, Logan, Utah, USA) to better assess the environmental conditions related to greenhouse gases dynamics. Sensors (9 each for temperature and O_2_) were inserted horizontally in the pile to about 1 m of depth and distributed in 9 locations at three heights (0.5 m, 1.0 m and 1.5 m) equidistantly along the pile. Half-hour average values were reported during the entire composting process. Both the temperature and O_2_ sensors were connected to a data logger (CR-1000, Campbell Scientific, Logan, Utah, USA). Millivolt outputs were converted to O_2_ concentration by first correcting it by the local temperature obtained in the pile and then using a linear regression obtained during lab calibration. The pile was turned weekly with an industrial compost turner. Prior to turning, all four gas sampling towers and buried sensors were removed from the pile area. Immediately after turning, we replaced the sensors and relocated the towers in the exact same position by using permanent ground markers (i.e., plastic stakes with a small circular flat plate at ground level) hammered in the soil at the beginning of the experiment. This assured consistency in the anemometer angle position with respect to wind direction.

Compost samples (approximately 1 kg each) were collected weekly at each of the 9 sites within the pile both pre- and post- turning and placed in 1-gallon Ziplock freezer bags (n = 18 samples weekly). Samples were stored at 4 °C and analyzed within 24 h after collection. Compost moisture content was determined on 10 g samples gravimetrically after drying at 105 °C for 24 h. Moisture units were expressed as g H_2_O on a gram of dry compost basis (g H_2_O.g^−1^). Bulk density was determined by adding compost up to a 100 mL volume mark in a beaker and oven drying the sample at 105 °C to constant weight. Bulk density units were g of dry compost per cm^3^ of volume (g cm^−3^). Compost pH was measured in a slurry with 3 g of fresh compost in 5 ml of D.I. water using a pH electrode (Denver Instruments, Bohemia, New York, USA)^45^.Ammonium (NH_4_^+^) and nitrate (NO_3_^-^) were measured after extracting approximately 3.5 g fresh compost in 75 mL of 2 M KCl and analyzed on a colorimetric discrete analyzer (Seal Analytical, Inc. Mequon, WI, USA, Model: AQ300); NO_3_^-^ was determined by cadmium reduction using the Griess-Ilosvay method, and NH_4_^+^ was determined by the indophenol blue method^46^ Inorganic N concentrations units were expressed per g oven dry compost at 65 °C (μg N·g^−1^). Potential net nitrification and N mineralization rates were determined by incubating approximately 3.5 g of compost in the dark for 7 d. The former was determined by differences in pre- and post- incubation ${\text{NO}}_{{3}}^{ - }$ concentration and the latter by the difference of the sum of ${\text{NH}}_{{4}}^{ + }$ and ${\text{NO}}_{{3}}^{ - }$ pre and post incubation using the procedure described above^47^. Total C and N were determined on dry, ground samples (SPEX Samples Prep Mixer Mill 8000D, Metuchen, New Jersey, USA) by elemental analysis (Carlo Erba Elantech, Lakewood, New Jersey, USA) using atropine as a standard and corroborating linearity by measuring the standard every 10 samples^47,48^. Compost porosity was determined in samples collected at three heights in the center part of the pile. We weighed compost samples (5 replicates/height location) in a 100 mL of volume, followed by the flask tare, deionized (D. I.) water addition to the 100 mL mark, and finally the recording the water mass. The compost and D.I. water mass difference was used to calculate the volume of the pore space in the original sample^49^.

The measured greenhouse gas fluxes were used to determine the greenhouse gas EF derived from composting (GHG EF_c_) according to Eq. (4):

4 $${\text{GHG EF}}_{{\text{c}}} = \frac{{\mathop \sum \nolimits_{t = 0}^{n} FluxGHG_{t} \times C_{f} \times BA_{p} \times t}}{{m_{fw} { }}}$$

where, ${\mathrm{GHG EF}}_{\mathrm{c}}=$ greenhouse gases emission factor derived from the turned compost pile (kg GHG-C or -N/ton of feedlot wet or dry), ${FluxGHG}_{t}=$ median daily greenhouse gases flux (kg m^−2^ d^−1^); ${C}_{f}$= conversion factor for expressing greenhouse gases as C or N; for CH_4_ we used 0.75, for CO_2_ we used 0.27, and for N_2_O we used 0.64; $t$= time interval, d (d); ${BA}_{p}$= ground base pile area (m^2^); and ${m}_{fw}$ = mass of wet or dry feedstock (food waste composted or total compost (Mg)). The EF values are hard to compare across the literature because of differences in methodologies, lack of equivalent units (e.g., wet versus dry compost), and the lack of data reported (e.g., duration of study). In this work we express our EF values in multiple units to facilitate comparison across studies and are presented as averages and median values (we show median values reflecting minimum and maximum, to account for methodological boundary conditions: fetch distances from > 5 to > 13 m long, see methods section and supplementary material).

Open-source statistical software ‘R^50^’ was used for greenhouse gas fluxes calculations (by means of Eq. 1) and for data filtering mentioned in supplementary material. To evaluate variability in physicochemical properties (pH, inorganic N, porosity, bulk density, gravimetric water content, gas concentrations, N mineralization, N nitrification, C:N, temperature, and O_2_) in the pile (top, middle and bottom) we performed two-way ANOVA using JMP Pro 16 (SAS Institute, Cary, North Carolina, USA). When data were not normally distributed, nonparametric statistics were applied for variable comparison by Spearman's rank correlation. Emissions factors were estimated in ‘R’ by integrating the median daily greenhouse gas values over the composting period using a spline function. The median integral (g m^−2^) was multiplied by the pile volume and divided by the pile fetch (15 m) to obtain the final median amounts emitted during composting. Statistical significance was defined as *p* < 0.05. Data are presented in the text as means and standard errors unless otherwise noted.

## Results

### Net greenhouse gases fluxes to the atmosphere

**Figure 2:**
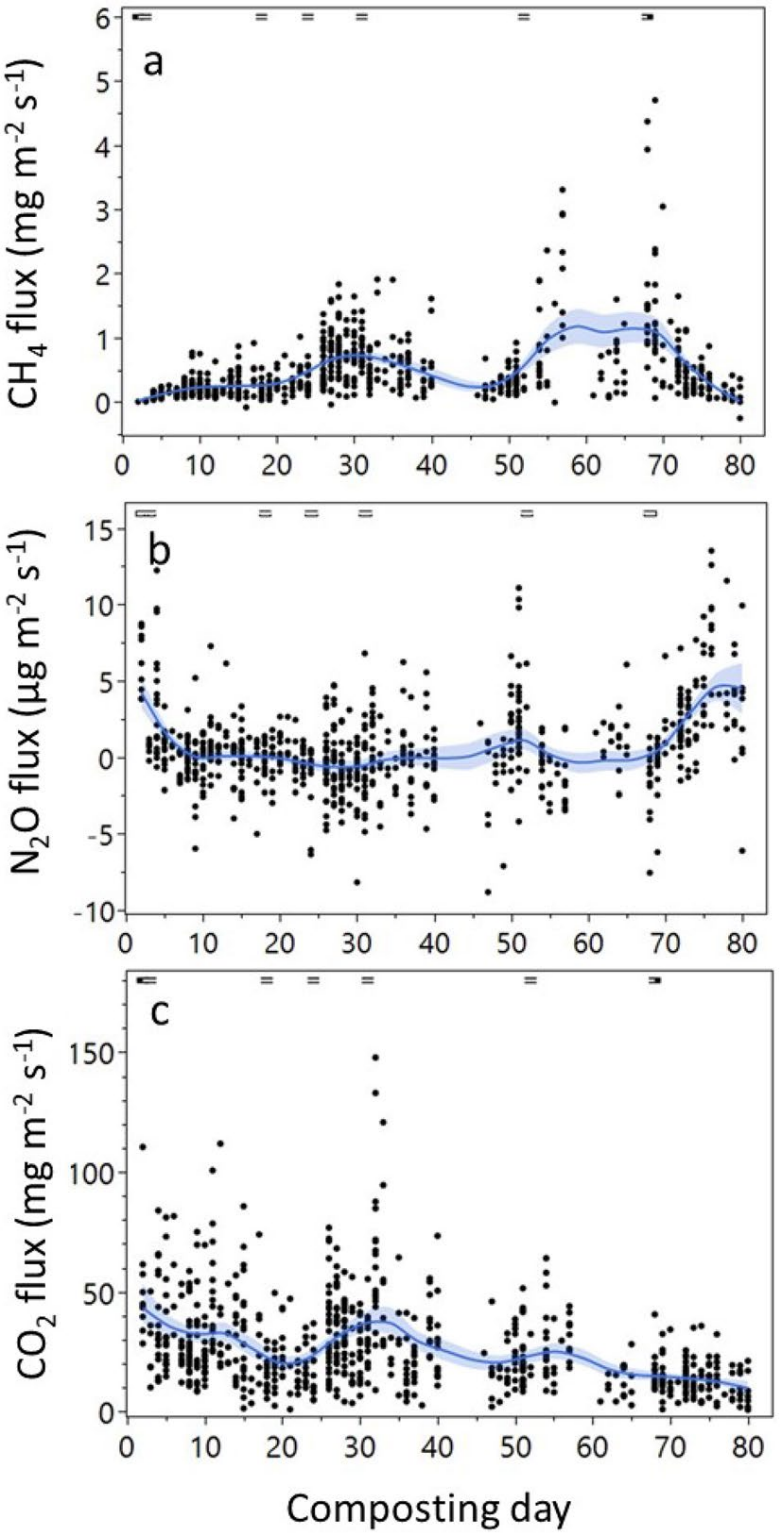
Food waste compost pile GHG fluxes during the composting period: CH_4_ (mg CH_4_ m^−2^ s^−1^) (**a**), N_2_O (μg N_2_O m^−2^ s^−1^) (**b**) and CO_2_ (mg CO_2_ m^−2^ s^−1^) (**c**). The dashed lines at the top of each graph represent the water addition d. Smoothed blue lines represent mean and confidence intervals calculated using spline function (λ < 0.05).

During the initial mesophilic phase (first 3 d of composting) CH_4_ emissions were low and CO_2_ and N_2_O emissions peaked (Fig. 2). Once the temperature exceeded 60 °C (thermophilic phase starting on day 5, Fig. 2a) CH_4_ emissions increased over time up to 4.7 mg CH_4_ m^−2^ s^−1^, after which they declined sharply on day 70 (during maturation phase). Watering events corresponded to periods of high CH_4_ emissions, with three peaks from d 28 to 35, and on day 57 and 69, yielding emissions of 1.9, 3.3 and 4.7 mg CH_4_ m^−2^ s^−1^, respectively. The largest values were found after the last two watering events on d 52 and 66 (2659 and 5243 L, respectively) (Fig. 2a). The highest fluxes of N_2_O (between 12 and 14 µg N_2_O m^−2^ s^−1^) occurred after initial watering events (7571 and 1893 L on day 1 and 4, respectively), on day 52 after watering, and between day 71 and 80 (Fig. 2b). Most of the N_2_O measurements during the study were below the detection limit of the instrumentation (<2 µg N_2_O m^−2^ s^−1^).

Carbon dioxide fluxes ranged from 0.8 to 148 mg CO_2_ m^−2^ s^−1^ (Figure 2c). Overall, the largest fluxes occurred during the first 3 d of the experiment. In general, higher fluxes were found prior to day 38 and declined progressively thereafter. Water additions did not result in significantly higher CO_2_ fluxes.

### Physicochemical and biogeochemical variables

**Figure 3:**
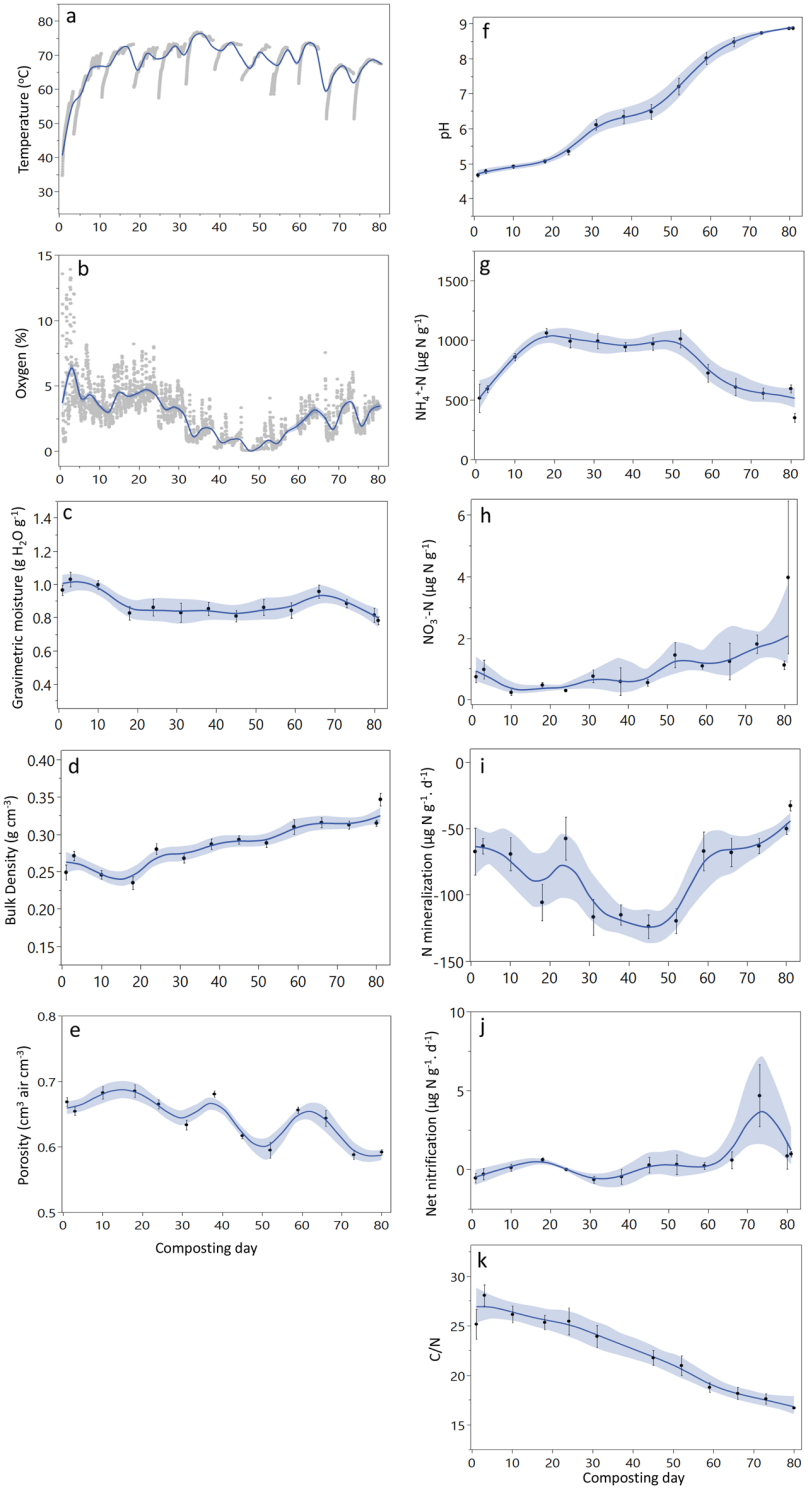
Physicochemical variables measured during composting: (**a**) temperature, (**b**) oxygen, (**c**) gravimetric water content, (**d**) bulk density, (**e**) porosity, (**f**) pH, (**g**) NH_4_^+^ concentration, (**h**) concentr ation, (**i**) net N mineralization, (**j**) net nitrification and (**k**) C:N ratio. Calculated mean curve and shaded confidence intervals were determined with smoothing spline function (λ = 0.05).

Compost temperatures quickly entered the thermophilic phase. Temperature was lower during the initial (1 to 20 d) and cooling down (60 to 80 d) periods ranging from 63.9 ± 0.10 °C (n = 8163) to 67.2 ± 0.06 °C (n = 7929), respectively (*p* < 0.001). Compost temperatures were higher between d 20 to 60 (70.8 ± 0.05 °C, n = 15465) (*p* < 0.001) (Fig. 3). Temperature was highest in the middle and lowest depths of the pile, and in the upwind location (*p* < 0.001). The lowest mean temperatures were found in the center location of the pile (*p* < 0.001). Compost O_2_ concentrations ranged from 0 to 15% and were highest during the first 20 d of the composting process (mean of 4.28% ± 0.04, n = 8163), then decreased to minimum values between d 40 to 60 (mean of 0.68% ± 0.01, n = 7212). Oxygen concentrations progressively increased until the end of the experiment (*p* < 0.05) (Fig. 3). The upwind and center locations of the pile had similar O_2_ concentrations, and the downwind location of the pile was generally more reduced (*p* < 0.001); O_2_ concentrations also decreased from the top to the bottom depths of the pile (*p* < 0.001).

Moisture decreased progressively after each water addition. Mean water content ranged from 0.45 to 0.5 g H_2_O g wet compost^−1^. Higher water contents were found at the top of the pile and after watering and turning events (*p* < 0.05 and *p* < 0.001, respectively). In general, both temperature and O_2_ concentrations decreased sharply a few hours after watering and/or turning and progressively increased as the pile dried. Compost pH increased dramatically, following a linear trend over time (R^2^ = 0.96, *p* < 0.001) from 4.7 at the beginning until it stabilized at approximately 8.7 during the last three weeks of the composting process (Fig. 3). Compost bulk density values increased significantly over time (R^2^ = 0.38, *p* < 0.0001) and porosity (pre-turning) decreased with time (R^2^ = 0.39, *p* < 0.0001) from about 0.7 to 0.6 (Fig. 3); there were no statistically significant patterns in bulk density or porosity with location in the pile.

Inorganic N was dominantly comprised of ${\text{NH}}_{{4}}^{ + }$. Ammonium concentrations increased rapidly from 280 µg ${\text{NH}}_{{4}}^{ + }$–N g^−1^ up to 1297 µg ${\text{NH}}_{{4}}^{ + }$–N g^−1^ over the first 10 d; high values were maintained between day 10 to 52 (Fig. 3). Ammonium concentrations started to decline after day 59 with a final average value of 352 ± 36 µg N g^−1^ on day 80. Nitrate concentrations were very low throughout the composting process with many samples below the analytical detection limit (< 0.05 ppm N). Higher ${\text{NO}}_{{3}}^{ - }$–N values were found during the last two weeks of the composting process (up to 18.1 µg ${\text{NO}}_{{3}}^{ - }$–N g^1^ on d 73 and 80) averaging 4.0 ± 2.5, µg N g^−1^, significantly higher when compared to the rest of the composting process (*p* < 0.0001). Most N mineralization rates were negative (94%, n = 209) and in 81% of those, ${\text{NH}}_{{4}}^{ + }$–N concentrations decreased by more than 50% during the incubation period. Net N mineralization averaged − 106.3 ± 5.3 µg N g^−1^ d^−1^ between d 18 and 52 (Fig. 3). Before and after that period, net N mineralization rates averaged − 65 ± 4.1 µg N g^−1^ d^−1^ (n = 107). Net nitrification rates were below the analytical detection limit until the last two weeks of the composting period when values were detectable but low (3.3 ± 1.4 µg N g^−1^ d^−1^, n = 27). The C:N ratio decreased during the composting period with the highest values found during the first three d (27.1 ± 0.95, n = 9) and a final value of 16.9 ± 0.3 (n = 3) (Fig. 3k). The same trend was observed for total C and N concentrations ranging from 25.7 ± 1.0 and 0.96 ± 0.04 (n = 9) during the first three d to 20.5 ± 0.5 and 1.21 ± 0.01 (n = 3) at the end of composting, respectively.

**Figure 4:**
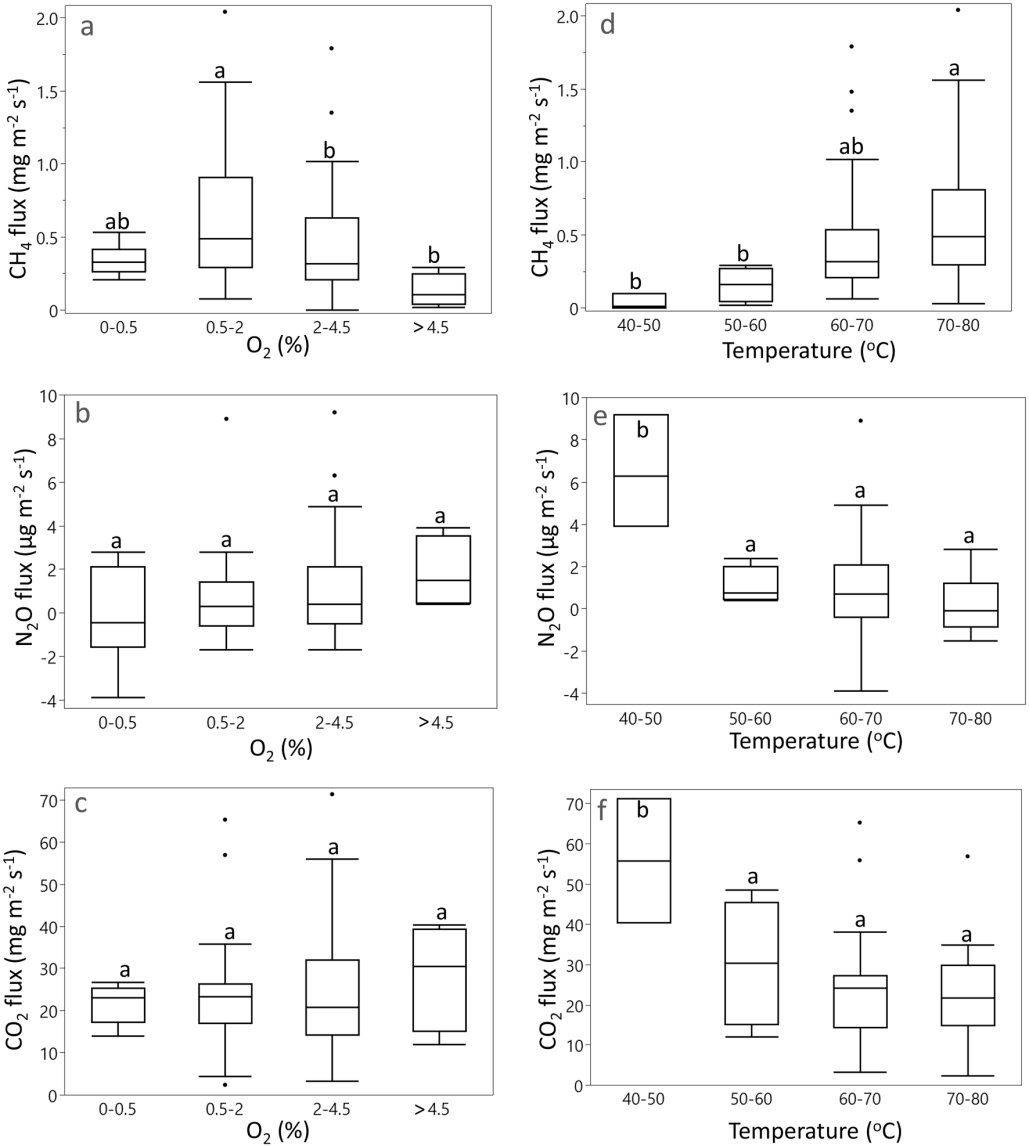
Daily mean GHG fluxes at different O_2_ concentrations and temperatures. Central line is the mean and top and bottom side of box plot are 75 and 25 quantile values. Statistically significant differences are marked with letters (*p* < 0.05).

High GHG flux events were correlated with specific temperature and redox conditions (Fig. 4). Daily mean CO_2_ and N_2_O fluxes were highest when compost temperature was between 40 °C and 50 °C (*p* < 0.0001) very early in the experiment, while the highest CH_4_ fluxes occurred at 60 to 80 °C (*p* < 0.05) later in the experiment. Daily mean CH_4_ fluxes occurred when O_2_ concentrations were between 0.5% and 2% (*p* < 0.05) whereas mean CO_2_ and N_2_O fluxes were not directly correlated with O_2_ (Fig. 4). There were also relationships between mean daily GHG fluxes and specific physicochemical parameters (Table 2). For example, CO_2_ fluxes were negatively correlated with pH, bulk density, and NO_3_–N, whereas N_2_O mean fluxes were positively correlated to ${\text{NO}}_{{3}}^{ - }$–N and inversely correlated to ${\text{NH}}_{{4}}^{ + }$ (Table 2). Daily CH_4_ fluxes, showed a slight positive correlation only with pH and bulk density and negative correlation with N_2_O fluxes (Table 2).

**Table 2:** Statistical multivariate analysis of GHG daily mean fluxes with physicochemical variables.

(**a**) Multivariate analysis correlation coefficient (r).													
	Compost day	O_2_ mean (%)	Temperature mean (°C)	CO_2_ flux (mg/m^2^s)	CH_4_ flux (mg/m^2^s)	N_2_O flux (µg/m^2^s)	Gravimetric moisture (g H_2_O/g)	Bulk density (g/ cm^3^)	pH	NH_4_–N (µg N/g)	NO_3_–N (µg N/g)	N min (µg N/g. day)	N nit (µg N/g. day)
Compost day	1												
O mean (%)	− 0.40*	1.00											
Temperature mean (°C)	0.27*	− 0.41*	1.00										
CO_2_ flux (mg/m^2^s)	− 0.63*	0.13	− 0.46*	1.00									
CH_4_ flux (mg/m^2^s)	0.37*	− 0.26*	0.29*	− 0.05	1.00								
N_2_O flux (µg/m^2^s)	0.18	0.11	− 0.44*	0.08	− 0.41*	1.00							
Gravimetric moisture (g H_2_O/g)	− 0.49*	0.54*	− 0.63*	0.36*	− 0.21	0.24	1.00						
Bulk density (g/cm^3^)	0.99*	− 0.43*	0.20	− 0.58*	0.38*	0.21	− 0.42*	1.00					
pH	0.99*	− 0.35*	0.20	− 0.62*	0.37*	0.22	− 0.38*	0.99*	1.00				
NH_4_ − N (µg N/g)	− 0.49*	− 0.25*	0.43*	0.24	− 0.01	− 0.57*	− 0.45*	− 0.53*	− 0.58*	1.00			
NO_3_ − N (µg N/g)	0.80*	0.01	− 0.08	− 0.49*	0.05	0.50*	− 0.13	0.78*	0.83*	− 0.81*	1.00		
N min (µg N/g.day)	0.20	0.60*	− 0.37*	− 0.18	− 0.09	0.47*	0.56*	0.19	0.29*	− 0.86*	0.65*	1.00	
N nit (µg N/g.day)	0.79*	0.13	− 0.02	− 0.56*	0.14	0.40*	− 0.06	0.75*	0.82*	− 0.80*	0.94*	0.74*	1.00

(**b**) Significance values (p) of GHG daily mean fluxes with physicochemical variables.													
Compost day	< 0.0001												
O mean (%)	< 0.001	< 0.0001											
Temperature mean (°C)	< 0.05	< 0.0001	< 0.0001										
CO_2_ flux (mg/m^2^s)	< 0.0001	0.2595	< 0.0001	< 0.0001									
CH_4_ flux (mg/m^2^s)	< 0.001	< 0.05	< 0.01	0.6653	< 0.0001								
N_2_O flux (µg/m^2^s)	0.1074	0.3504	< 0.0001	0.4804	< 0.001	< 0.0001							
Gravimetric moisture (g H_2_O/g)	< 0.0001	< 0.0001	< 0.0001	< 0.01	0.0674	< 0.05	< 0.0001						
Bulk density (g/cm^3^)	< 0.0001	< 0.0001	0.0793	< 0.0001	< 0.001	0.065	< 0.0001	< 0.0001					
pH	< 0.0001	< 0.005	0.0771	< 0.0001	< 0.001	0.0462	< 0.0005	< 0.0001	< 0.0001				
NH_4_ − N (µg N/g)	< 0.0001	< 0.05	< 0.0001	< 0.05	0.9169	< 0.0001	< 0.0001	< 0.0001	< 0.0001	< 0.0001			
NO_3_ − N (µg N/g)	< 0.0001	0.9238	0.4828	< 0.0001	0.6661	< 0.0001	0.2374	< 0.0001	< 0.0001	< 0.0001	< 0.0001		
N min (µg N/g. day)	0.0683	< 0.0001	< 0.001	0.1114	0.4491	< 0.0001	< 0.0001	0.0921	< 0.01	< 0.0001	< 0.0001	< 0.0001	
N nit (µg N/g. day)	< 0.0001	0.2451	0.8813	< 0.0001	0.2294	< 0.001	0.5911	< 0.0001	< 0.0001	< 0.0001	< 0.0001	< 0.0001	< 0.0001

Asterisk indicates negative and positive r correlation values larger than 0.25.

## Discussion

### Greenhouse gas emissions from composting and associated drivers

The MMB approach adapted for a three-dimensional geometric structure allowed us to measure whole-system greenhouse gas fluxes continuously throughout the entire composting process without any disturbance to the pile surface. High frequency measurements of GHG fluxes and physicochemical parameters enabled us to identify drivers of GHG emissions in real time and to identify shifts in biogeochemical dynamics during the composting process. Pile CO_2_ fluxes were higher when temperatures were lower (Table 2a, Fig. 4f). The overall inverse correlation of CO_2_ fluxes with temperature suggests that heterotrophic respiration is a dominant process at the beginning of the composting process. This is supported by the large increase in NH_4_^+^ concentrations during the first week (Fig. 3g). More than 40% of organic matter is generally degraded during the first week of composting when temperatures are < 60°C^14^. Nitrous oxide fluxes also occurred at the beginning of the composting process. The high N content of food waste could potentially promote large N_2_O emissions during decomposition^26,51–54^. The above-mentioned rapid increase in NH_4_^+^ concentrations in the initial phase along with lower temperatures (25–50 °C on d 1–3) provided optimal conditions for autotrophic (at temperatures < 40 °C) and heterotrophic nitrification (at temperatures > 40 °C)^51^. The latter is more likely to be the prevalent microbial process potentially responsible for N_2_O production in the compost pile^55^ given the temperature range recorded (> 40 °C) (Fig. 3a). The overall nitrification reaction releases hydrogen ion (H^+^), which likely contributed, along with the fermentation process, to the low pH values found in the beginning of the experiment^20^. This is supported by the overall inverse relationship between N_2_O and CO_2_ fluxes and pile temperature (Table 2a) and the highest measured fluxes found at a temperature range of 40 to 50 °C (Fig. 4e,f). As the thermophilic phase established, N_2_O fluxes decreased to levels below the detection limit of the analytical instrumentation. This was consistent with unsuitable conditions for ammonia- and nitrite-oxidizing bacterial growth (low O_2_ concentration and high temperature) or enhanced N_2_O consumption via denitrification (Fig. 4e)^52^. The consistently high ${\text{NH}}_{{4}}^{ + }$ concentrations during the thermophilic phase suggests that ${\text{NH}}_{{4}}^{ + }$ was not consumed until later in the experiment when temperatures started to decrease, and nitrifying/denitrifying processes were more likely to occur (Fig. 3g).

We found that CH_4_ emissions were relatively low during most of the composting process and increased as a result of watering events, high temperature, and low O_2_ availability. As opposed to CO_2_ and N_2_O fluxes, the highest CH_4_ fluxes were found at 60 to 80 °C and 0.5 to 4.5% O_2_ (Fig. 4a,d). This is consistent with thermophilic conditions favoring decomposition during a period of sustained low O_2_ concentrations (likely providing the anaerobic conditions necessary to support methanogenesis)^53^. Also, the fact that high CH_4_ fluxes always occurred after watering events indicate that, in conjunction with low O_2_ concentrations, moisture played a significant role in enhancing CH_4_ fluxes particularly during the thermophilic phase. Substrate availability was likely to be high during this phase. During the early phases of decomposition, easily degradable organic matter releases organic and inorganic acids that can decrease pH^54–56^. Low pH has been associated with acidogenic fermentation of labile carbohydrates and fats, which can produce volatile fatty acids (VFAs) such as acetic, propionic and butyric acids among others, as well as alcohols^56,57^. We measured low pH at the beginning of the experiment suggesting VFA formation could have occurred and subsequently became available to methanogens as O_2_ declined^58,59^. Substrate pH increased during the thermophilic phase (Fig. 3f) towards the optimal pH conditions for the growth of methanogens^60^ and, corresponded to higher CH_4_ emissions (Fig. 2a). The high ${\text{NH}}_{{4}}^{ + }$ concentrations found throughout the thermophilic phase may have also contributed to enhanced CH_4_ emissions via inhibition of CH_4_ oxidation^61^.

The continuous net negative N mineralization rates during most of the composting process was an important finding and is an indicator of ${\text{NH}}_{{4}}^{ + }$ immobilization (Fig. 3i). Enhanced microbial activity and N assimilation was likely related to the significant labile C present in food waste^62^. It is also possible that abiotic ammoxidation of cellulose or lignin (oxidative C conversion with ${\text{NH}}_{{4}}^{ + }$) occurred. At high temperatures (> 70 °C), cellulose and lignin can be decomposed to monosaccharides, which react with ${\text{NH}}_{{4}}^{ + }$ to form long-chain amino sugars, sugar acids, and imidazoles^63^. It is also possible that ${\text{NH}}_{{4}}^{ + }$ was incorporated into humic-like substances^64,65^ which can be more prevalent at the end of the composting process^66^. This last process might be responsible for the immobilization of large amounts of ${\text{NH}}_{{4}}^{ + }$ from day 50 to the end of composting, when ${\text{NH}}_{{4}}^{ + }$ concentrations dropped by half and the mean C:N ratio significantly decreased to values < 16 (Fig. 3i). The fact that N mineralization rates were progressively less negative from day 50 to the end of the composting process suggests that ${\text{NH}}_{{4}}^{ + }$ assimilation occurred via the above-mentioned processes, consumption via nitrification, and/or NH_3_ volatilization given the increase in pH. Further compost chemical characterization by ^15^N- and ^13^C- NMR spectrometry and measurements of NH_3_ emitted during compost process would facilitate the quantification of N dynamics during compost-related decomposition. A key result here is that the food waste composting process did not appear to produce large amounts of NO_3_^-^ (values at least an order of magnitude smaller than those found for ${\text{NH}}_{{4}}^{ + }$, Figs. 3g,h) that could both drive higher N_2_O emissions and pollute local water resources.

These findings suggests that fine tuning the composting process might further reduce GHG fluxes. For example, smaller and more frequent watering events (e.g., weekly before turning) during the initial to the thermophilic phase would likely yield sufficient moisture content (~ 0.50 to 0.65%w/w H_2_O) while minimizing O_2_ consumption^12^. Turning events stimulate aerobic conditions which favors CH_4_ oxidation and reduces methanogenesis; turning also lowers the likelihood of high temperatures that hamper microbial activity and reduce the quality of the compost^12,67,68^. Reducing watering events towards the end of the composting process could minimize the formation of anaerobic conditions, particularly favorable at this time where the highest bulk density (Fig. 3d) and the lowest porosity (Fig. 3e) are found. This would decrease both ${\text{NO}}_{{3}}^{ - }$ consumption and N_2_O emissions and enhance compost N content and quality. This would also lower the EF for the entire composting process given the high global warming potential of N_2_O.

### Emission associated with composting food waste

We calculated median EF values of 6.6 to 8.8 kg CH_4_–C/Mg wet FW, 0.010 to 0.013 kg N_2_O-N/Mg wet FW and 441 to 596 kg CO_2_/Mg wet FW (Table 3, see supplementary material for details regarding the range). Our median CH_4_ EF of 8.8 kg CH_4_–C/Mg wet FW was chosen as the more representative EF for the compost pile (value derived for fetch values > 13 m, see supplementary material). This CH_4_ EF is similar to the value reported for food waste composted in static aerated piles of municipal waste treatment plants in Germany using a gradient concentration method (up to 8.6 kg CH_4_–C/Mg wet FW, Table S2 supplementary material)^69^, but larger than other results where static and dynamic chambers were used (see Table S2). Estimates of EF values are likely affected by the experimental approach, particularly pile size, measurement frequency, type of bulking material, food waste/bulking material ratio and composting time length. We found generally larger EF values in studies performed at a facility scale^29,69^. A recent study compared a similar MMB approach and the dynamic chamber method in a green waste turned windrow compost pile and found that the dynamic chamber method EF estimates were always smaller, with a discrepancy of 40, 54 and 244% for CO_2_, CH_4_ and N_2_O, respectively^37^. Most of the previous studies of GHG EF estimates from food waste composting have used smaller size piles, laboratory incubations, static chambers or large open dynamic tunnel with total enclosure (see Table 2S for reference). All of these methodological approaches have limitations for capturing both the composting process under facility-scale pile conditions and inherited interference related to each applied method. To our knowledge, no previous study of food waste composting has been done at a facility scale measuring high frequency GHG fluxes as reported here. Thus, it is possible that the current approach was able to better capture total fluxes during the composting process.

**Table 3:** GHG Emission factors intercomparison among studies with the MMB method and landfill estimates.

Emission factor units	CH_4_		CO_2_		N_2_O		kg CO_2_eq/ton dry feedstock^b^	Reference
	Mean ± SE^a^	Median	Mean ± SE^a^	Median	Mean ± SE^a^	Median		
kgGHG-C or-N/Mg dry compost	5.90 ± 0.73	5.14	105.64 ± 9.30	95.14	0.008 ± 0.005	0.008	217	This work
kgGHG-C or-N/Mg wet feedstock^c^	3.08 ± 0.38	2.68	55.20 ± 4.86	49.71	0.004 ± 0.003	0.004		
kgGHG-C or-N/Mg wet food waste	10.1 ± 1.24	8.8	181.0 ± 15.93	163	0.014 ± 0.009	0.013		
kgGHG-C or-N/Mg dry food waste	25.3 ± 3.11	22.0	452.5 ± 39.82	407.5	0.023 ± 0.014	0.022	926	
kgGHG-C or-N/Mg dry compost	3.18		169		0.000318182		115	^37^
kgGHG-C or-N/Mg dry compost	2.90 ± 0.60		12.0 ± 2.3				105	^36^
kg GHG-C or -N/Mg dry food in landfill^d^	41 to 161		323.1^e^				1487 to 5832	^6,70,71^
kg GHG-C/Mg dry compost in landfill^f^	28.30		216.9				1026	

^a^Mean ± standard error, n = 716, EF derived from daily average flux calculated with fetch distance > 5 m (see Figure S5, supplementary material).

^b^Including only CH_4_ and N_2_O, using global warming potential values of 27.2 and 278 for CH_4_ and N_2_O, respectively.

^c^Total weight of wet organic matter used (48.2 metric tons composed of 14.7 t of food waste and 28.1 t of yard debris).

^d^Range of published non collected CH_4_ EF from landfilling food waste.

^e^Value from Lee et al^6^.

^f^Emission factor estimates if the feedstock used in this work compost was landfilled. For this calculation wastes moisture content values were derived from IPCC^72^ (60% for food waste and yard debris) and CH_4_ EF from landfilling food waste from Lee et al^6^.

In landfills, food waste generally dominates CH_4_ production given the large first-order decay rate constant (k) (0.7 yr^−1^) for CH_4_ production, which is at least three times larger than for other organic solid waste (green waste, paper and wood)^9^. Consequently, food waste is the feedstock with the largest CH_4_ EF in landfills, with values ranging from 41 to 161Kg CH_4_–C/Mg dry FW^6,70,71^. Our CH_4_ EF values from composting food waste are 38 to 84% lower than those found in landfills using published estimates (Table 3) and 79% smaller if the entire composition of the compost studied here (food waste + yard debris) was disposed in a landfill. Thus, while CH_4_ and N_2_O were detected from composting, the overall GHG emissions were much lower than they would be for the counterfactual fate of landfilling this material. The results from this study are most closely comparable with two previous studies that used a somewhat similar MMB method. This study’s CH_4_ EF value of 5.90 ± 0.73 kg CH_4_–C/Mg dry compost was almost twice as large as those found for garden waste (3.18 kg CH_4_–C/Mg dry compost^37^) and manure and green waste composting (2.90 ± 0.60 kg CH_4_–C/Mg dry compost^36^) (Table 3). The larger values found in our study are consistent with the larger labile C source found in food waste in comparison to that found for green and manure wastes, as well as differences in the associated C and nutrient concentrations, and the length of the sampling period.

The state of California is planning to manage 16.3 MMT of organic waste by 2025. Recent legislation (SB 1383) aims to recover at least 20% of the edible food by the same year^27^. If we assume the current proportion of food waste (44%) is the same by the year 2025 and discount a 20% diversion to feed Californians in need, 5.7 MMT of food waste would need to be managed. If this material is composted, a GHG reduction potential of 1.4 to 11.2 MMT CO_2_e could be achieved by in 2025 when compared to landfilling food waste. This represents a 39 to 84% CH_4_ emissions reduction (Table 4).

**Table 4:** Expected GHG reduction from food waste compost management for year 2025 in the state of California.

Management type	Emission Factor (KgCO_2_e/ton dry waste)	California expected food waste for 2025 (MMT dry weight)^b^	Total GHG emission (MMTCO_2_e)	Percentage of CH_4_ reduction relative to landfilling food waste	Reference
Compost^a^	925.9	2.3	2.1		This work
Landfill (non-collected CH_4_ emissions)	1523.2 5832	2.3 2.3	3.5 13.3	39.2 84.1	^6^ ^71^
Net GHG reduction potential			1.4^c^ and 11.2^d^		

^a^Emission factor including only CH_4_ and N_2_O derived emissions.

^b^Net amount of expected food waste for year 2025 (5.7 MMT) dry weight calculated assuming food waste moisture content of 60%.

^c^Using Lee et al^6^ CH_4_ emission factor from food waste landfilling.

^d^Using Wang et al^71^ CH_4_ emission factor from food waste landfilling.

## Conclusions

Food waste is a large source of GHG emissions in the waste sector^6^. Here, we reported one of the most comprehensive commercial scale whole pile studies of GHG emissions and associated drivers during food waste composting. Using the MMB approach, we found higher GHG EFs than less comprehensive measurement methods. This is likely because the MMB approach provided much higher resolution data from the continuous, whole pile assessment of GHG fluxes than less frequent measurements in space and time provided by other methodologies. Even though the EFs were higher than previous studies, we found that food waste composting resulted in 39 to 84% lower CH_4_ emissions than landfilling. Pile CH_4_ emissions were higher after wetting events during the thermophilic phase of composting and at the end of the process. Turning served to aerate the pile and temporarily lower CH_4_ emissions. Pile N_2_O emissions were detected at the beginning and end of the composting process but were mostly below the method’s detection limit. The pattern in N_2_O fluxes likely reflected the more optimal conditions for organic matter decomposition including lower temperatures and high substrate availability at the start of the experiment, and cool temperatures, high moisture, and low redox conditions near the end of the process. Persistent low NO_3_^-^ availability, the primary substrate for denitrification, likely contributed to low overall N_2_O emissions. Our results suggest that increasing the pile aeration and decreasing watering amount or frequency, especially in the middle and end of the composting process could potentially further lower CH_4_ emissions. We show that GHG emissions from food waste composting are lower than landfilling and suggest that future deployment of continuous measurement approaches such as the one described here can help further lower emissions and contribute to climate change mitigation.

## Supplementary Information

Supplementary Information 1.

Supplementary Information 2.

## Data Availability

Data is available upon request to T. Pérez.
