# Supplementary material for: Assessing the climate change mitigation potential from food waste composting
Source: Sci Rep. 2023 May 10;13:7608. doi: 10.1038/s41598-023-34174-z (PMC10172324; doi:10.1038/s41598-023-34174-z)
Supplement: Supplementary file 1 — Supplementary Information 1. [file 41598_2023_34174_MOESM1_ESM.docx]

**Supplementary material for manuscript: “Assessing the climate change mitigation potential from food waste composting.”**

**Tibisay Pérez, Sintana E. Vergara, and Whendee L. Silver.**

**Micrometeorological mass balance method details and data filtering steps**

*Data analysis for flux determinations*

For each 8-minute period, we filtered out values using the following criteria: 1) When the average 1-min wind direction and windspeed variation were less than 30^o^, 60^o^ and 90^o^ and 3 m/s among the 8 anemometers, respectively; 2) when fetch values were smaller than a minimum threshold (< 5 m in our case); 3) when wind direction was perpendicular to a tower pair gas collection axis during an 8-min cycle; 4) when wind direction originated from the other compost facility windrow piles (90^o^ to 220 ^o^); 5) when wind direction was outside permissible wind speed thresholds (1.5 to 8 m/s) and 6) when turbulence intensity in the downwind tower was significantly larger than that of upwind tower.

*Fetch distance calculations and exclusion criteria*

Fetch is defined as the distance an air mass travels from an upwind to a downwind tower in the compost pile at a given wind direction. The pile had a rectangular base and the towers were placed symmetrically facing the pile along the sides (Figure S1); therefore, the fetch length depended on the specific orientation of each tower. The fetch distance calculation was done with trigonometric functions of two asymmetrical right triangles for T2 and T4 located widthwise, and two symmetrical right triangles for T1 and T3 located lengthwise (Fig. S1). For a given wind direction, downwind towers had a fetch > 0 and a fetch = 0 for upwind towers (Figure S2). Fetch was calculated by averaging wind direction values at the four top anemometers over the 8-minute flux measurement periods to minimize the effect of short-term wind direction fluctuations^1, 2^. Method testing and data quality assessments were performed to filter data (see below).

To facilitate fetch calculations the towers were placed 1 m away from the pile and they were aligned symmetrically with the tower from the opposite side of the pile. Therefore, the equations used for fetch calculation are the same for towers 1 and 3 and for towers 2 and 4. Figure S1 illustrates the pile dimensions (to scale) and inclination angle (225^o^) orientation. The amplitude of the inclination angles at each side of the right triangle (α and α’ for towers 2 and 4 and β and β’ for towers 1 and 3) were used to determine the fetch distance across the 15 m pile length (Figure S1). For tower 1 and 3, the right angle was located along the 15 m section of the pile (225^o^ and 45^o^ or tower 1 and 3, respectively) and β and β’ angle amplitudes were used to determine the fetch distance across the 4 m pile width (Table S1). Fetch values allowed us to identify upwind and downwind towers. For a given wind direction, the downwind towers had fetch > 0 and for those upwind towers had fetch = 0 (Figure S4 shaded area).


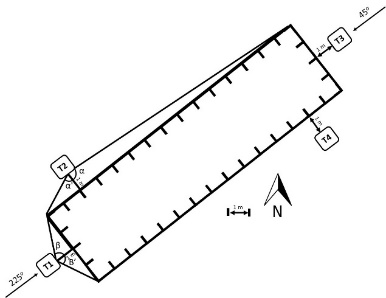


Figure S1. Compost pile dimensions and tower locations and orientation.

Table S1. Fetch equations per tower determined from trigonometric functions and angles described in Figure S1.

| Wind direction from Tower 1 (degrees) | Wind direction from Tower 3 (degrees) | Inclination angles  (β and β’) | Fetch (m) equation | Equations for tower |
| --- | --- | --- | --- | --- |
| 341 to 38 | 161 to 218 | 64 to 7 | $\left( (2-\tan\beta)\div\sin\beta) \right)$ | 1 and 3 |
| 39 to 45 | 219 to 225 | 6 to 0 | $\left( 16\div\cos\beta)-(1\div\cos\beta\right)$ | 1 and 3 |
| 45 to 51 | 226 to 231 | 0 to 6 | $\left( 16\div\cos\beta')-(1\div\cos\beta' \right)$ | 1 and 3 |
| 52 to 108 | 232 to 228 | 7 to 64 | $\left( (2-\tan\beta^{'})\div\sin\beta') \right)$ | 1 and 3 |
| Wind direction from Tower 2 (degrees) | Wind direction from Tower 4 (degrees) | Inclination angles  (α and α’) | Fetch (m) equation | Equations for tower |
| 229.6 to 248.96 | 49.6 to 68.96 | 85.6 to 68.96 | $\left( 13\div\sin\alpha)-(1\div\cos\alpha\right)$ | 2 and 4 |
| 249 to 157.91 | 69 to 337.92 | 66 o 22.92 | $\left( 5\div\cos\alpha)-(1\div\cos\alpha\right)$ | 2 and 4 |
| 158 to 198.44 | 338 to 18.44 | 23 to 66.44 | $\left( 2\div\sin\alpha')-(1\div\cos\alpha' \right)$ | 1. and 4 |

*Elimination of the back diffusion effect and data filtering*

A minimum average wind speed is required at the topmost anemometers to minimize back diffusion effects that can potentially lead to overestimated fluxes^2^. Greenhouse gas concentrations were examined along the vertical profile of sampling inlets to test the method assumption that no vertical flux should exceed the upper most sampling height (3.5 m). This implies that at the highest sampling port GHG concentrations between upwind and downwind towers should be no larger than background atmospheric concentrations. We found that GHG concentrations decreased with increasing sampling height and the values at the highest position were indistinguishable from background atmospheric concentrations (Figure S2). The predominant wind direction ranged from 210^o^ to 315 ^o^, thus T3 and T4 were in the prevailing downwind positions (Figures S2 c, d, g, h, k and l). Wind directions from 0^o^ to 90^o^ were infrequent in the dataset (6,800 data points out of 107,102, 6.3%), and thus T1 contributed very little to the downwind data (Figure S2a and e). When T2 was downwind, GHG concentrations from the upwind tower (T4) were higher than the atmospheric background (Figure S2 d, e and f) reflecting the contamination of our signal from nearby feedstock and compost piles. Therefore, we filtered all data that had a wind direction between 90^o^ and 220^o^. A trend of higher N_2_O values at lower tower sampling ports was found for the average N_2_O concentrations for the downwind towers (Figure S2 c, f, i and j), however, the difference among the values found in the upwind towers were indistinguishable in most cases. This implied a higher N_2_O detection limit in comparison with the other GHG measured. Wind properties showed distinctive airflow patterns at the prevailing downwind towers (T3 and T4). We calculated 8-minute flux intervals when GHG Δ*C_3.5m_* data at the highest ports (3.5 m) exhibited background air values with their respective uncertainties (0.2 ppm, 5 ppm and 0.01 ppm for CH_4_, CO_2_ and N_2_O, respectively).


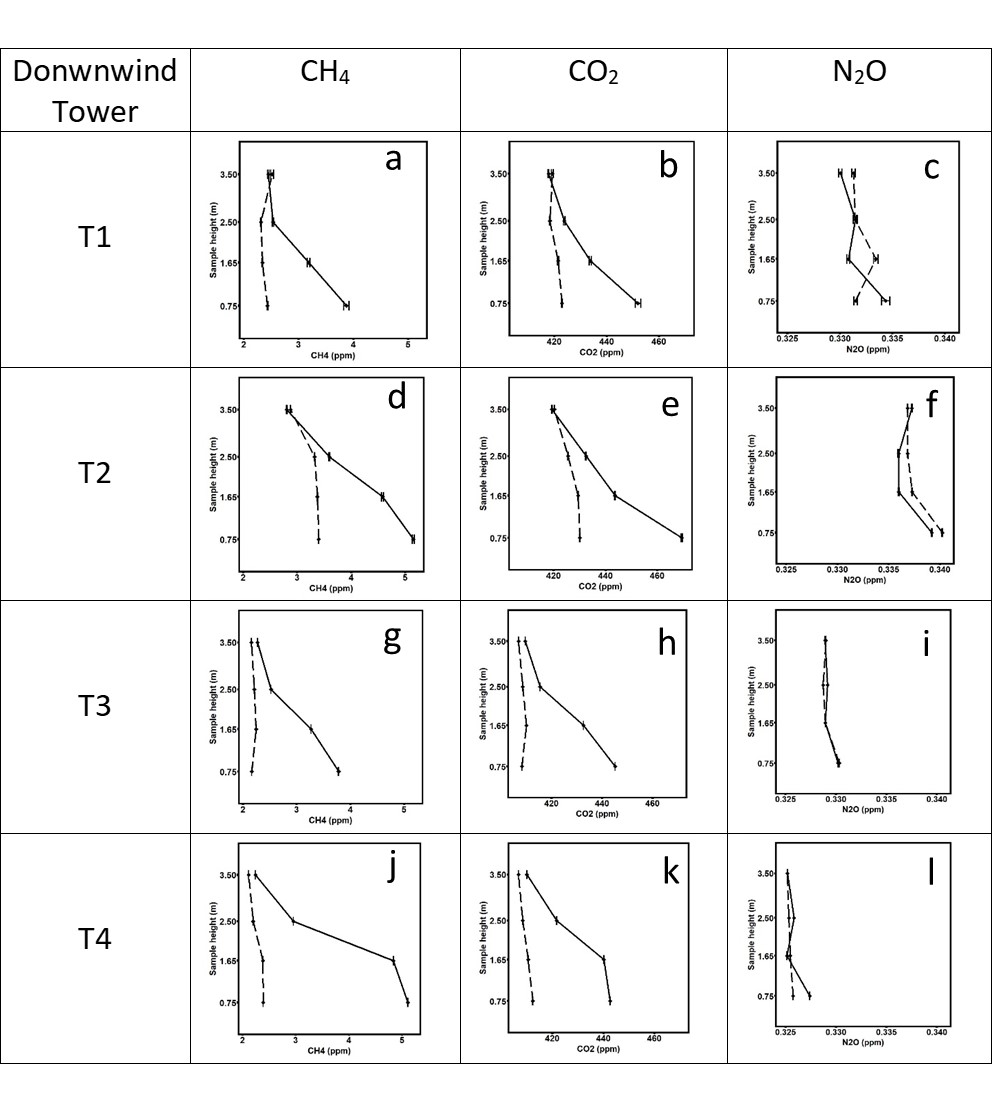


Figure S2. CH_4_, CO_2_ and N_2_O average (±se) concentrations as a function of height for upwind (dashed line) and downwind (solid line) towers when T1 (a to c), T2 d to f), T3 (g to i) and T4 (j to l) are in the downwind position.

We found that wind speed can affect back diffusion effects and potentially overestimate fluxes, as did Wagner-Riddle et al^2^. The mean GHG ΔC*_3.5 m_* at the top anemometers was inversely correlated with mean wind speed (Figure S3). We found that a minimum of 1.5 m/s of wind speed at the top anemometers had Δ*C* values equivalent to background air GHG uncertainty. This minimum windspeed was thus used as a threshold to identify potential a back-diffusion effect. This data filter yielded 3324 fluxes for the 80-day sapling period.

*Effect of pile shape on airflow*

Further filtering had to be applied related to the pile shape effect. The original micrometeorological mass balance method was developed for circular manure tanks with surfaces that were at ground level^2^. However, typical compost windrows are a triangular pyramid with a rectangular base. Thus, we needed to account for the effect of the three-dimensional geometric shape on the flux determination. To do this we oriented the pile lengthwise along the axis of the prevailing winds (determined from prior wind measurements at the facility). We placed one tower pair aligned lengthwise and the other widthwise maximizing the fetch distance for each tower pair (see Figure S1). We evaluated how pile shape influenced the airflow by measuring wind speed, wind direction and turbulence intensity shifts from the upwind to the downwind tower in the specific lengthwise and widthwise prevailing wind directions. Turbulence intensity, defined as the ratio of the root-mean-square of wind fluctuations to the mean wind speed, is an indication of potential eddy and gust formation in an air mass. If pile shape did not affect air flow, these variables would be expected to be equal between upwind and downwind towers.

Wind speed in both downwind towers increased with height (Figure S3 a and e) but higher values were found for both upwind and downwind tower positions in the lengthwise tower pair. The difference between the upwind and downwind wind speed in the widthwise tower pair (0.18 to 0.48 m/s) was larger in comparison to the lengthwise pair (< 0.12 m/s; *p* < 0.05, t-student), suggesting that the pile obstructed airflow more effectively when air masses encounter the larger surface area in the widthwise direction. Wind direction differences per height increment between upwind and downwind towers in the lengthwise tower pair ranged from 22^o^ to 33^o^, whereas for the widthwise tower pair was between 36^o^ to 71^o^ (Figure S3 b and f). This implied that some of the air mass from the widthwise direction traveled around the pile, as opposed to a more linear trajectory between the two widthwise towers. These wind direction shifts can potentially produce a larger fetch distance than the expected straight-line calculated with the micrometeorological mass balance method. This airmass behavior has been observed in computational fluid dynamics modeling around a rectangular body volume. The wider the geometry of the transverse width, the larger the flow distortion at the top and side faces will be^2, 3^. There were no statistically significant differences between average turbulence intensity per height in the upwind and downwind towers of the lengthwise tower pair at any height, whereas 20% larger values were found in the downwind tower of the widthwise tower pair (*p* < 0.05, student-t, Figure S3 c and g).


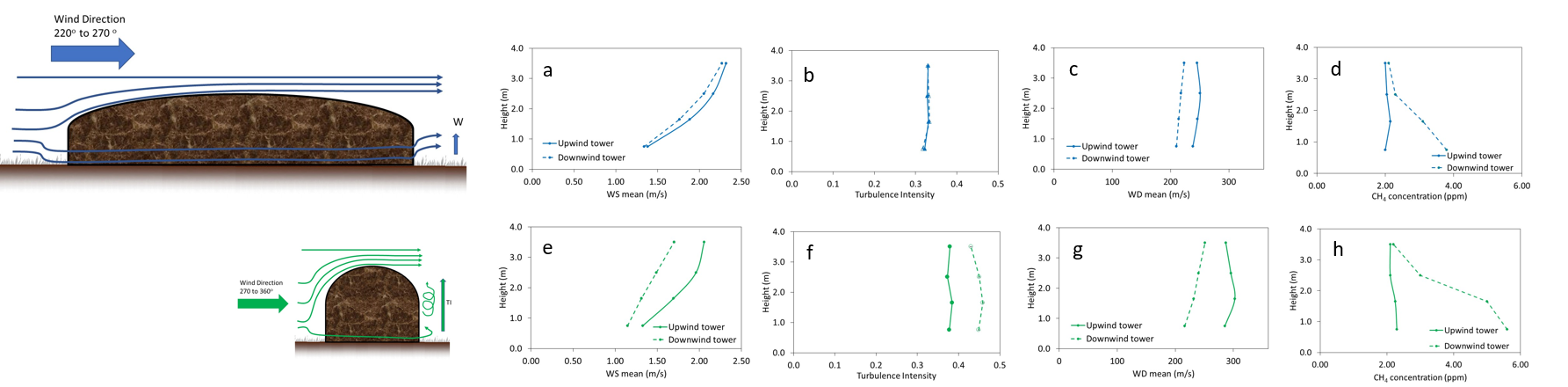


Figure S3. Average ± s.e. of wind speed (a, e), turbulence intensity (b, f), wind direction (c, g) and methane concentrations (d, h) versus height of upwind (dashed lines) and downwind (solid lines) towers from widthwise (270 to 360^o^, circles) and lengthwise (220 to 270^o^, triangles) prevailing wind directions.

The lower windspeeds experienced by the widthwise tower pair led to a larger wind direction diversion, and larger turbulence intensity than the lengthwise tower pair. This suggests that the GHG concentrations measured in the widthwise direction were more likely to be enhanced with height due to the large probability of eddy wind circulation. We found between 2- and 4-times higher CH_4_ concentrations in the three lower heights for the widthwise tower pair (Figure S3 d and h). A similar pattern, but with less concentration enhancement, was observed for CO_2_ (figure S2 h and k). Therefore, pile shape significantly affected GHG fluxes when measured at the widthwise tower pair, but not when using the lengthwise towers.

*Effect of minimum fetch screening on GHG fluxes and Emission Factors (EF)*

Carbon dioxide and CH_4_ fluxes were not normally distributed (*P* < 0.0001). We found an inverse polynomial correlation between mean CO_2_ (degree 2, r^2^ = 0.91, *P* < 0.0001) and mean CH_4_ (degree 3, r^2^ = 0.77, *P* < 0.0003) fluxes with fetch distance. Theoretically, GHG fluxes determined by the micrometeorological mass balance method should not be correlated with fetch distance because GHG ΔC measured at each height is expected to be directly proportional to the fetch distance travelled over the pile. Our observation implies that when fetch distances were short, flux measurements may have been more susceptible to airflow effects derived by the pile shape. We found that wind direction differences between upwind and downwind towers for the lengthwise tower pair increased when fetch values were smaller than 5 m (Figure S4 a). In contrast, for the widthwise tower pair WD differences between upwind and downwind towers were much larger for all the fetch values (Figure S4 b). This effect can be observed in the GHG EF calculated with screening data with different minimum fetch values (>1, >3, >5, >7, >9, >11 and >13 m). EF values were larger when data with small fetch values were included in GHG flux determination (Figure S5). These results imply that fluxes calculated with fetch values < 5 m would be overestimated. Thus, our fluxes were screened for fetch distance > 5 m and those data were used to calculate EF. We also used the GHG fluxes daily median values to minimize the effect of outliers in the mean daily values and reported a range of EF values calculated with data screened for fetch distances > 5m to > 13m (Figure S5). Thus, after applying all the above-mentioned filtering, our final GHG flux data reported here refer only to data collected from the lengthwise tower pairs (with T3 as downwind tower), wind speed at the top anemometers >1.5 m/s and fetch distance values > 5m. These filter set yielded a total of 713 valid fluxes.


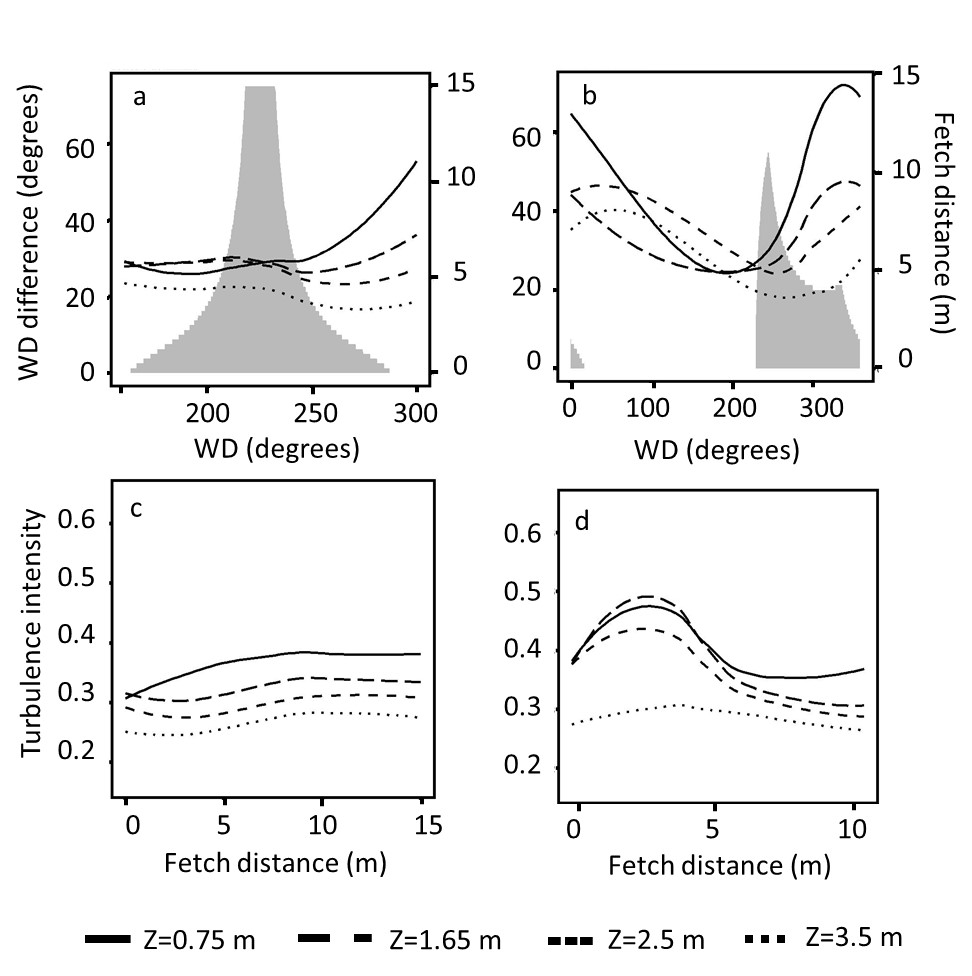


Figure S4. Lengthwise (T3 downwind) (a) and widthwise (T4 downwind) (b) tower pairs fetch distance values (border of shaded gray area) and WD differences between upwind WD and downwind WD tower pair as a function of wind direction at each height (z). Turbulent intensity values as a function of fetch distance at downwind towers T3 (c) and T4(d). Lines represent height where wind direction differences and turbulence intensity values were measured: 0.75 m (—), 1.65m(‒ ‒), 2.5 m (---) and 3.50 m (···).


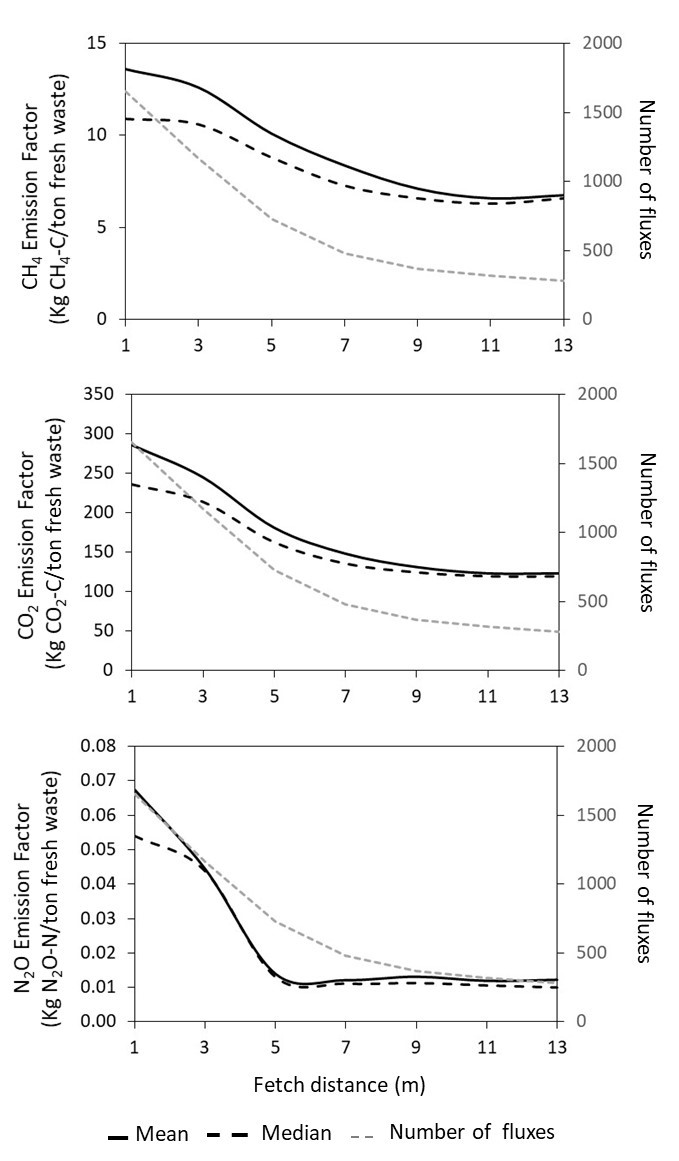


Figure S5. Greenhouse gas emission factors from the 11-week composting period derived from data screening with minimum fetch distance values (>1, >3, >5, >7, >9, >11 and >13 m).

*Method recommendations to minimize data loss by filtering.*

The following recommendations must be considered to minimize data loss by filtering. First, it is advisable to place the pile in an open area and as far as possible from other GHG sources. Second, because pile shape largely affects flux determinations in the widthwise pile axis, we recommend placing only two towers in the lengthwise axis oriented with the prevailing wind direction. Third, place the downwind tower at a distance where turbulence intensity is not different from the upwind tower to minimize the effect of eddies formed as the air masses exit the pile. Fourth, the farther the downwind tower is from the pile, the taller it needs to be so that it can capture the entire GHG plume. Finally, monitor turbulence intensity values at each height for both towers during the first week of the experiment to assess if the downwind tower placement is appropriate (they should not be statistically different; if so, the downwind tower should be placed farther away from the pile).

Table S2. Greenhouse gases fluxes and emission factors from food waste composting found in the literature. (See larger table in the attached excel file).

Experimental studies references:

^1, 4-14^

References

1. Kent, E. R., Bailey, S. K., Stephens, J., Horwath, W. R. & Paw U, K. T. Measurements of Greenhouse Gas Flux
from Composting Green-Waste Using Micrometeorological Mass Balance and Flow-Through Chambers. *Compost Science & Utilization*, 1-20 (2019).

2. Wagner-Riddle, C., Park, K. & Thurtell, G. W. A micrometeorological mass balance approach for greenhouse gas flux measurements from stored animal manure. *Agricultural and Forest Meteorology* **136**, 175-187 (2006).

3. Lee, Y. T., Boo, S. I., Lim, H. C. & Misutani, K. Pressure distribution on rectangular buildings with changes in aspect ratio and wind direction. *Wind and Structures* **23**, 465-483 (2016).

4. Y He *et al*. Nitrous Oxide Emissions from Aerated Composting of Organic Waste. *Environmental science & technology* **35**, 2347-2351 (2001).

5. Colon, J. *et al*. Determination of the energy and environmental burdens associated with the biological treatment of source-separated Municipal Solid Wastes. *Energy Environ. Sci* **5**, 5731-5741 (2012).

6. Chan, Y. C., Sinha, R. K. & Wang, W. Emission of greenhouse gases from home aerobic composting, anaerobic digestion and vermicomposting of household wastes in Brisbane (Australia). *Waste management & research* **29**, 540-548 (2011).

7. Amlinger, F., Peyr, S. & Cuhls, C. Green house gas emissions from composting and mechanical biological treatment. *Waste Management & Research* **26**, 47-60 (2008).

8. Beck-Friis, B., Pell, M., Sonesson, U., Jönsson, H. & Kirchmann, H. Formation and Emission of N_2_O and CH_4_ from Compost Heaps of Organic Household Waster. *Environ Monit Assess* **62**, 317-331 (2000).

9. Clemens, J. & Cuhls, C. Greenhouse gas emissions from mechanical and biological waste treatment of municipal waste. *Environmental Technology* **24**, 745-754 (2003).

10. Adhikari, B. K., Trémier, A., Barrington, S., Martinez, J. & Daumoin, M. Gas emissions as influenced by home composting system configuration. *Journal of Environmental Management* **116**, 163-171 (2013).

11. Scheutz, C., Samuelsson, J., Fredenslund, A. M. & Kjeldsen, P. Quantification of multiple methane emission sources at landfills using a double tracer technique. *Waste management* **31**, 1009-1017 (2011).

12. Phong, N. T. Greenhouse Gas Emissions from Composting and Anaerobic Digestion Plants., 1-109 (2012). https://bonndoc.ulb.uni-bonn.de/xmlui/bitstream/handle/20.500.11811/5130/3002.pdf?sequence=1&isAllowed=y

13. Hellmann, B., Zelles, L., Palojarvi, A. & Bai, Q. Emission of Climate-Relevant Trace Gases and Succession of Microbial Communities during Open-Windrow Composting. *Applied and Environmental Microbiology* **63**, 1011-1018 (1997).

14. Hobson, A. M., Frederickson, J. & Dise, N. B. CH_4_ and N_2_O from mechanically turned windrow and vermicomposting systems following in-vessel pre-treatment. *Waste management* **25**, 345-352 (2005).
